# Sexuality and psychological well-being in different polycystic ovary syndrome phenotypes compared with healthy controls: a cross-sectional study

**DOI:** 10.1186/s12905-022-01983-9

**Published:** 2022-09-25

**Authors:** Fatemeh Bahadori, Shahideh Jahanian Sadatmahalleh, Ali Montazeri, Malihe Nasiri

**Affiliations:** 1 Department of Midwifery and Reproductive Health, Faculty of Medical Sciences, Tarbiat Modares University, Tehran, Iran; 2 Population Health Research Group, Health Metrics Research Center, Iranian Institute for Health Sciences Research, ACECR, Tehran, Iran; 3 Department of Biostatistics, Faculty of Medical Sciences, Shahid Beheshti University, Tehran, Iran

**Keywords:** Sexual function, Quality of life, Phenotypes of polycystic ovary syndrome, Anxiety and depression

## Abstract

**Introduction:**

Polycystic ovary syndrome (PCOS) is the most common endocrine disorder in women of reproductive age. The present study aimed to compare the women with different PCOS phenotypes with the healty group in terms of sexual function, depression, anxiety and quality of life scale.

**Materials and methods:**

The present cross-sectional study was carried out on 192 women with PCOS (classified on the basis of Rotterdam criteria into four categories) and 50 healthy controls. All participants were asked to fill out the valid and reliable questionnaires of FSFI (Female Sexual Function Index), HADS (Hospital Depression and Anxiety Scale) and SF-12.

**Results:**

In the HADS questionnaire, phenotype B achieved the highest mean score in anxiety and depression domains, whereas, phenotype B had the lowest mean score in the FSFI and SF-12 quassionnaires. Furthermore, there was a significant difference between the women with PCOS phenotypes and the control grroup in arousal, lubrication, pain, and mean total score of FSFI (P < 0.05). In regression logistic analysis, age, infertility and depression were predictors of sexual dysfunction (P < 0.05).

**Conclusion:**

The results indicated significant differences in terms of sexual dysfunction, depression, anxiety and quality of life in the women suffering from different phenotypes of PCOS compared with the healthy group. These results provide evidence that care and recommendations for improving women’s QoL and sexual function should be considered according to the relevant PCOS phenotypes.

## Introduction

Polycystic ovary syndrom (PCOS) is the most common endocrine disorder in women of reproductive age. The estimated prevalence of PCOS in different population is 5–24% [1, 2]. PCOS is charactrized by large ovaries, menstural irregularities, clinical and biochemical hyperandrogenism.

The clinical signs as hirsutism, acne, alopecia and seborrhea along with obesity and infertility may cause a significant amount of emotional distress [3, 4]. These physical, physiological and psychological changes can cause mood disturbances, including a significant reduction in quality of life, lower self- esteem, marital and social maladjustments [2, 5].

Sometimes it can cause high levels of anxiety and tension that lead to depression, eating disorders and sexual dysfunction [3].

Sexual functioning and response in women is a complicated psychobiosocial phenomenon and is affected by multiple factors. In PCOS women, the factors affecting sexual functioning include infertility, deranged hormone levels especially androgens, obesity and associated problems like metabolic syndrome, body image issues and low self—esteem [6].

Some studies have reported that PCOS can impair women’s sexual dysfunction [5]. But there are several studies that have not confirmed the sexual function impairment in patient with PCOS. The results are paradoxical as various studies have provided that, these women have gained similar score as normal women in sexual function test [7–9].

Quality of life based the perception of ife and is quite subjective [10], also it is a multidimensional concept including physical, psychological and social aspects of health [11]. But based on the definition provided by The Word Health Organization, quality of life can be defined as a state of complete physical, mental and social wellbeing and four dimensions are considered for it including: physical health, psychological aspect, social contacts and social environment [12].

Studies have shown quality of life is under influence of health status especially as chronic diseases have undesirable impacts on individual’s social, psychological and physical status [13]. Some studies have reported quality of life these women are lower than of normal women [14, 15].

However, it seems that different phenotypes of PCOS in terms of hormonal, anthropometric and metabolic indices are different [16], there are limited researches to assess metabolic profiles of these diagnostic groups [17]. Hence, it is not completely apparent that these phenotypes suffer from the same negative psychlogical health risks or not. It is important to know more about HRQoL and psychological well-being in PCOS women to develop strategies and interventions to enhance their HRQoL.

In addition, to our knowledge there is currently no research comparining psychological aspects of different PCOS phenotypes with the control group in Iranian population. Acording to above objectives the following hypotheses were derived and studied in PCOS patients and the the control group.

Hypotheses: sexuality and psychological well-being are different in phenotypes of PCOS and there is significant differnce between different PCOS subgroups and the control group.

## Materials and methods

This cross- sectional study included five comparative groups ( four phenotype groups) and one control group (lack of PCOS). Upon approval of the Medical Ethics Committee of Tarbiat Modares University (IR.MODARES.REC.1397.153), the study was performed on 242 sexually active participants aged (18–40 years) (85% of PCOS women were infertile and 60% obese women). The study group included women with PCOS referred to an infertility clinic in Arash Hospital in Tehran Province, Iran from May 2018 to February 2019. Also the control group included the patients referred to Arash Hospital, consistent with healthy fertile married women, who were non-pregnant, non-breastfeeding (parity $\ge$ 1) without PCOS (or other major gynecological conditions, e.g. endometriosis or chronic disease) and had a regular menstrual cycle. They were selected from the patients’companions by convenience. At first, sample size was calculated based on taking 95% confidence interval and 85% power to test.

Then, using the appropriate formula at 95% confidence interval and error 3 (d). It was found that a sample size of 215 women was needed with 10% sample loss:

$${\text{Sample}}\;{\text{size}}\;{\text{estimation}}\;{\text{formula}}:n = \frac{{\left( { z_{{1 - \frac{\alpha }{2}}} } \right) s^{2} }}{{d^{2} }}.$$

**Fig. 1:**
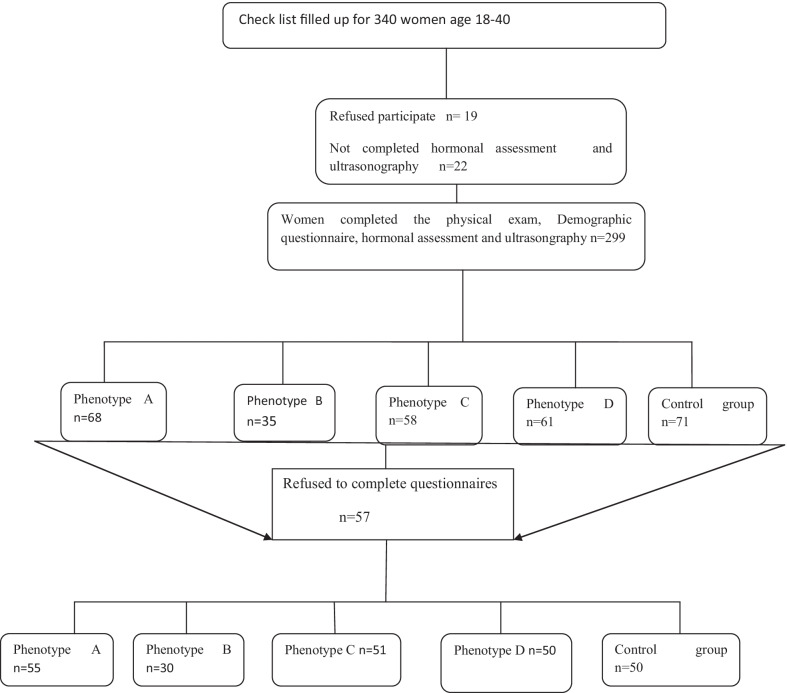
Study population flowchart

The study flowchart for the present study is presented in Fig. 1. By excluding women with incomplete data (women who refused participate n = 19, or not completed hormonal assessment and ultrasongraphy n = 22 or women who refused to fill out the questionnaires) n = 57. There were 98 people in total.

Finally, 55 patients with phenotype A, 30 patients with phenotype B, 56 patients with phenotype C, 51 patients with phenotype D, study.

The presentation of PCOS can be categorized into separate phenotype, depending on the features used in the diagnostic criteria.There are four phenotypes of PCOS acocording to Rotterdam criteria [18].

Although the NIH 2012 evidence-baced metthodolog PCOS Workshop recommends the use of broder Rotterdam diagnostic criteria, with definite reporting of specific four phenotypes in all reserch studies and clinical care to maximize the homogeneity and comparability in research and clinical initatives [19]. This approach was also confirmed by the 2018 international evidence-bacsed guidline for the assessment and management of PCOS [20]. This guidline confirms the Rotterdam PCOS diagnostic criteria in adults (two of clinical or biochemical hyperandrogenism, ovulatory dysfunction, or polycystic ovaries on ultrasound) and where irregular menstrual cycles and hyperandrogenism are present, ultrasound is not necessary in diagnosis. Within eight years of menarch, both both hyperandrogenis and ovoulatory dysfunction are required, ultrasound not recommended. Acording to the description provided, these phenotypes include: phenotype A consists of hyperandrogenism, ovulatory dysfunction and polycystic ovarian morphology (PCOM), phenotype B is composed of hyperandrogenis and ovulatory dysfunction, phenotype C consists of hyperandrogenism and PCOM, and phenotype D consists of ovulatory dysfunction and PCOM.

Phenotypes A and B are more associated with obesity, metabolic dysfuncyion and menstural irregulrities [21].

Disttribution of PCOS phenotype is variable and dependent on how the population was identified. Lizneva et al. [21] carried out a meta-analysis study to detect PCOS phenotype prevalence (95% CI) in clinical versus unselected populations: The results were as follows: phenotype A: 50% (45–54%) versus 19% (13–27%), phenotype B: 13% (11–17%) versus 25% (15–37%). Phenotype C: 14% (12–16%) versus 34% (25–46%), phenotype D: 17% (13–22%) versus 19% (14–25%). Differences between clinical and unselected populations were statistically significant for phenotypes A, B and C [22].

The inclusion criteria were as follows: aged 18–40 years, married, non-pregnant, Iranian race, giving personal consent to enter the study, For the PCOS group, completion of primary school as the minimum level of education and no medication with hormones known to influence serum androgen levels, including anti-androgenic drugs and oral contraceptive pills during three months before the study were required. Due to the drug effect on the results, we should not include those PCOS patients who were consuming metformin due to PCOS (not because of the high blood glucose level). Those participants who were not eligible for the study such as patients with thyroid dysfunction, abnormal prolactin levels, congenital adrenal hyperplasia (CAH), Cushing’s syndrome (CS), androgen-secreting tumors, and diagnosed cardiovascular disease (CVD), as well as those taking oral contraceptives and anti-androgenic drugs were excluded from this study.

### Clinical and anthropometric assessment

All patients were examined for anthropometric parameters, menstrual cycles and hirsutism by a trained midwife, and were asked for demographic characteristics, as well as diagnosis and history of amenorrhea. Regularity of menstrual cycles was assessed in all patients. We described ovulation disorder as the menstrual cycle duration of more than 35 days or lack of lack of menstrual cycle for more than three months.

### Clinical hyperandrogenism was measured by

Modified Ferriman Gallwey (mFG), which was used for scroring the presence of terminal hairs over nine body areas. (ie. upper lip, chin, chest, upper and lower back,, upper and lower abdomen, things, and upper arms) from 0 to 4, and m-FG score ≥ 8 was considered hirsutism [23].

Anthropometric measurements, which were performed for all participants in this study, included body weight, height, and waist circumference measurements. Height and weight were scaled with the subjects in light clothes and without shoes. Waist circumference was evaluated using a flexible tape at the midline between the lower rib border and the curved superior border of the ilium (at the level of the umbilicus) at the end of normal exhalation whereas the participants were in the standing position. BMI was computed based on the World Health Organization (WHO) guidelines. Calculation formula was weight (in kilograms) divided by height squared (in meters) (kg/m^2^). We described ovulation disorder as the menstrual cycle duration of more than 35 days or lack of menstrual cycle for more than three months (oligo/anovulation).

### Measures

#### Demographic questionnaire

A checklist related to demographic features, including age, weight, height, BMI, waist to hip circumference ratio (waist/hip circumference), occupation status, educational level, infertility status, and medical history.

### FSFI

Female Sexual Function Index (FSFI) questionnaire is a validated test that evaluates all phases of the female sexual cycle in the past four weeks. The questionnaire consists of 19 items divided into six subscales (domains): sexual desire, sexual arousal, lubrication, orgasm, sexual satisfaction, and dyspareunia. Each item is rated on a scale from 0 to 5 or from 1 to 5, where 0 indicates no sexual activity in the past month. Scores for each of the six domains are calculated by summing up individual domain question scores and multiplying the result by the domain factor (i.e., 0.6 for desire, 0.3 for arousal and lubrication, and 0.4 for orgasm, satisfaction, and pain). The minimum possible score to achieve is 2 and the maximum is 36. A higher score in each domain indicates a better status [24]. Cutting point for the total scale and subscales were as follows: total scale 28, desire 3.3 arousal 3.4, lubrication 3.4, orgasm 3.4, satisfaction 3.8, pain 3.8, Scores greater than the cut point indicated good functioning [25]. The reliability and validity of this questionnaire have been confirmed in Iran [26].

### Quality of life (QoL)

The Short Form Health Survey (SF-12) includes 12 questions related to eight dimensions (sexual performance, physical role, physical pain, general health, energy and vitality, social performance, emotional role, and mental health), which in divided into two subscales of physical and mental health. The greatest score obtained for each section or subscale is 100 and the least score is zero with a higher score indicating a better health status [27]. The validity and reliability of this questionnaire have previously been confired in Iran [28].

### Anxiety and depression

The Hospital Anxiety and Depression Scale (HADS) is used to assess depression and anxiety. The instrument has two subscales including anxiety (HADS-A) and depression (HADS-D). It is a self-administered instrument consisting of 14 questions. The instrument has two subscales including anxiety (seven items) and depression (seven items). All items are rated from 0 to 3. Sum scores < 8 indicate normal range, scores 8–10 reflect mild alterations and scores ≥ 11 indicate clinical relevance of symptoms [29]. A study on the Persian version of the HADS has shown that this scale has satisfactory reliability and validity for measuring psychological symptoms in Iranian patients [30].

### Laboratory assessment

All participants in the present study underwent Transvaginal ultrasonography. Ovaries containing 12 or more follicles measuring 2–9 mm in diameter and/or enlarged ovarian volume (> 10 mm^3^) on abdominal ultrasonography were considered to have a positive polycystic sonographic view (Andocavity, 7.5 MHZ probe). Blood samples were also drawn for assessment of sex hormone-binding globulin (SHBG) and total testosterone (TT) levels. Free androgen index (FAI) was computed by TT (nmol/L)/SHBG (nmol/L) × 100) by commercial kits (Pars Azmoon Inc, Tehran, Iran) using Auto-analyzer BT2000 device. Biochemical measurement of TT and SHBG levels was performed based on the electro-chemiluminescence method (Demeditec Diagnostics GmbH. Lise-Meitner-Strabe 2.24145 Germany).

### Statistical analysis

Normal and non-normal quantitative variables were reported as Mean ± Standard Deviation (SD). Qualitative variables were presented as number (percentage). Primarily, the quantitative variables were checked for normality using Kolmogorov–Smirnoff’s (KS) test. One-way ANOVA was applied for the normal variables and Kruskal–Wallis (KW) test was used for the non-normal and ordinal variables. If there was a significant group effect, a pairwise comparison of the groups was performed using Mann–Whitney’s U test (MW). Then Bonferroni’s correction performance (P < 0.005) was considered significant. Qualitative variables were compared using the Chi-square test. In order to compare normal variables between two PCOS subgroups. Also, to examine the relashionship between sexual function and independent variables (quantitative and qualitative variables), binary logistic regression (cut-off point 28) was used. In this study, considering the cut-off point of 28 in terms of sexual function, the participants were divided into appropriate (> = 28) and inappropriate groups (< 28), and sexual function was considered as two state dependent variable. Considering this state, Binary logestic regression was used to control confounder variables and simultaneously examine the independent variablels. Regression logistic coefficient was calculated. Statistical significance was set at P < 0.05. The obtained data were analyzed by the SPSS software (Statistical Package for the Social Sciences, version 22.0, SPSS Inc., Chicago, IL, USA).

### Results

Table 1 shows an overview of the demographic characteristics of women with different phenotypes of PCOS and the control group. The age of the participants ranged from 18 to 40 years. As can be seen, there is a significant difference between the PCOS subgroups and the control group in terms of age, WHR, education level, number of children, number of abortionand infertility status (P < 0.05), but no significant difference is observed in occupation and BMI (P > 0.05).

**Table 1:** Comparison of demographic characteristics between different PCOS phenotypes and the control group

Variable	HA + PCOM + OD (A) n = 55	HA + OD (B) n = 30	HA + PCOM (C) n = 56	OD + PCOM (D) n = 51	Control (CO) n = 50	*P *value
Age (year)*	29.18 + 5.71	31.55 + 5.68	31.67 + 5.05	31.28 + 5.5	34.18 ± 4.13	< 0.001
BMI (Kg/m^2)^)*	27.21 + 4.80	26.59 + 3.85	25.60 + 3.67	26.45 + 3.75	26.96 ± 3.12	0.13
Number of children						< 0.001
0	45	17	42	37	0	
1	9	11	11	12	41	
2	1	1	3	2	9	
3	0	1	0	0	0	
Number of abortion						0.02
0	46	22	38	44	40	
1	5	5	12	5	10	
2	4	3	4	0	0	
3	0	0	2	2	0	
WHR*	0.87 ± 0.021	0.88 ± 0.023	0.87 ± 0.026	0.87 ± 0.016	0.85 ± 0.51	< 0.001
Occupation status**						0.11
Housewife	51 (92.7)	28 (93.3)	53 (94.6)	45 (88.3)	41 (82)	
Employed	4 (7.3)	2 (6.7)	3 (6.4)	6 (11.7)	9 (18)	
Educational level**						< 0.001
Primary	16 (29.09)	9 (30.01)	5 (8.93)	17 (33.33)	3 (6)	
Secondary	22 (40.01)	10 (33.33)	24 (42.85)	16 (31.37)	20 (40)	
Higher	17 (30.9)	11 (36.66)	27 (48.21)	18 (35.29)	27 (54)	
Infertility status**						< 0.001
Yes	47 (85.71)	25 (73.68)	48 (85.29)	43 (85.71)	0 (0)	
No	8 (14.28)	5 (26.31)	8 (14.7)	8 (14.28)	50n	

HA: Hyperandrogenism, OD: Ovulatory Dysfunction, PCOM: Polycystic Ovarian Morphology BMI: Body Mass Index. WHR: Waist Hip Circumference Ratio, PCOS: Polycystic Ovary Syndrome

* Values are given as mean ± SD using Kruskal–Wallis test

** Values are given as number (%) by using Chi-square test

Table 2 presents the statistics for FSFI in different phenotypes of PCOS and the control group. As shown, there is no statistically significant differnce between different PCOS subgroups and the control group in the scores of desire, orgasm and satisfacation (P > 0.05). However, there is a significant difference between the PCOS phenotypes and the control grroup in the mean total score of FSFI, arousal, lubrication and pain. In the arousal domain, in addition, there is a significant difference between the score of phenotype B and other categories and the control group in the arousal group (P < 0.05). In the pain domain, there are significant differences between the scores of phenotypes A, B, C and D and the control group, but in total score, there are significant differences between the scores of phenotypes A and B with the control group. Also, in the domains of arousal, lubrication and pain and total score, phenotype B expresses the lowest score and the control group expresses the highest score.

**Table 2:** Scores and total scores for the domain subgroups of FSFI between the different groups

Variable	HA + PCOM + OD (A) n = 55	HA + OD (B) n = 30	HA + PCOM (C) n = 56	OD + PCOM (D) n = 51	Control (CO) n = 50	*P *value
Desire	3.75 ± 0.85	3.48 ± 0.72	3.69 ± 0.94	3.69 ± 0.73	3.75 ± 0.77	0.642
Arousal	4.12 ± 0.87	4.51 ± .85	4.01 ± 1.04	4.08 ± 0.84	4.51 ± .85	0.002
Lubrication	4.69 ± 0.82	4.32 ± 1.15	4.63 ± 0.91	4.95 ± 0.99	4.89 ± 0.86	0.032
Orgasm	4.68 ± 0.903	4.106 ± 1.22	4.43 ± 1.02	4.66 ± 0.92	4.66 ± 0.912	0.192
Satisfaction	4.90 ± 1.02	4.45 ± 1.18	4.71 ± 1.14	4.95 ± 0.84	4.79 ± 0.905	0.313
Pain	2.99 ± 1.06	3.09 ± 1.206	3.17 ± 0.96	3.16 ± 1.26	6.59 ± 9.01	< 0.001
Total FSFI	25.16 ± 3.33	22.7 ± 4.2	24.66 ± 4.16	25.51 ± 3.29	28.78 ± 9.49	< 0.001
Sexual dysfunction*						
Yes	25 (45.5%)	16 (53.3%)	24 (42.8%)	21 (41.1%)	25 (50%)	< 0.001
No	30 (55.5%)	14 (46.6%)	32 (57.2%)	30 (58.9%)	25 (50%)	
Simultaneity of anxiety and sexual dysfunction	24 (96%)	16 (100%)	22 (91.6%)	21 (100%)	6 (24%)	0.830
Simultaneityof depression and sexual dysfunction	5 (20%)	10 (62.5%)	8 (33%)	7 (33%)	5 (20%)	0.001

HA: Hyperandrogenism, OD: Ovulatory Dysfunction, PCOM: Polycystic Ovarian Morphology PCOS: Polycystic Ovary Syndrome

*P *values refer to the Kruskal–Wallis test

*Values are given as number (%) by using Chi-square test

Anxiety and depression:

Table 3 compares the mean scores of different domains of HADS in different phenotypes of PCOS and the control group. In depression domain, there is a significant difference between subgroup B and the control group. Phenotye B with (8.43 ± 3.91) and the control group with (5.32 + 3.75) express the highest and the least scores, respectively. In anxiety domain, there is a significant difference between subgroups A and B and the control group. Phenotye B with (11.73 ± 3.39) and the control group with (8.46 + 3.78) express the highest and the least scores, respectively.

**Table 3:** Comparing the scores of HADS between different PCOS phenotypes and the control group

Variable	HA + PCOM + OD (A) n = 55	HA + OD (B) n = 30	HA + PCOM (C) n = 56	OD + PCOM (D) n = 51	Control (CO) n = 50	*P *value
Anxiety	10.74 ± 4.21	11.73 ± 3.39	10.5. ± 3.61	10.00 ± 4.13	8.46. ± 3.78	0.004
Depression	6.74 ± 3.73	8.43 ± 3.91	6.37 ± 3.78	6.31 ± 3.47	5.32. ± 3.7	0.02

HA: Hyperandrogenism, OD: Ovulatory Dysfunction, PCOM: Polycystic Ovarian Morphology PCOS: Polycystic Ovary Syndrome

*P* values refer to the Kruskal–Wallis test

### Quality of life (QoL)

Table 4 provieds the results obtained from summing up of the mean scores of SF-12 domains in diffferent PCOS phenotype categories. As shown, in the MCS subgruop, there is a significant difference between phenotypes A, B,C and D and the control group. In this domin, phenotype B expreeses the lowest score (39.11 ± 8.83) and the control group expresses the highest score (48.44 ± 8.84).

**Table 4:** Comparison of the SF-12 mean scores between different PCOS phenotypes and the control group

Variable	HA + PCOM + OD (A) n = 55	HA + OD (B) n = 30	HA + PCOM (C) n = 56	OD + PCOM (D) n = 51	Control (CO) n = 50	*P *value
MCS	41.70 ± 10.40	39.11 ± 8.83	41.40 ± 9.66	40.89 ± 10.18	48.44 ± 8.84	< 0.001
PCS	43.72 ± 7.75	43.66 ± 6.50	43.77 ± 7.51	45.73 ± 7.40	45.61 + 7.38	0.397

HA: Hyperandrogenism, OD: Ovulatory Dysfunction, PCOM: Polycystic Ovarian Morphology, PCOS: Polycystic Ovary Syndrome, SF-12: The 12-item Short Form Health Survey, PCS: Physical Component Summary, MCS: Mental Component Summary

Values are given as M ± SD using One-way ANOVA

Table 5 presents the relationship between sexual function and demographic variables in the study subjects. As shown, the odds ratio (OR) is not significant in P < 0.05 for BMI and WHR. The odds ratio (OR) for BMI is equal to 0.959 with the confidence interval (CI 95%) (0.864,1.064) and 1.018 for WHR with the confidence interval (CI 95%) (0.989,1.048). Because an OR higher than 1 in this study means that, no significant relashionship is observed between BMI,WHR and sexual function. However, the coefficients are significant for the age variable. The value of the odds ratio for age is equal to 1.085 with the confidence interval (CI 95%) (1.002,1.174). As a result, a significant relashionship is obserwed between age and sexual function, and with increasing one year of age, the chance of developing sexual dysfunction increases by 8.5%.

**Table 5:** Relationship between the participants’sexual function and demographic variables in logistic regression analysis

Variables	Beta	SE	*P *value	OR^*^	(95%CI)
Age	0.081	0.40	0.044	1.085	(1.002,1.174)
BMI	− 0.042	0.015	0.428	0.959	(0.864,1.064)
WHR	0.018	0.015	0.23	1.018	(0.989,1.048)

BMI: Body Mass Index, WHR: Waist Hip Circumference Ratio

* Regression Logistic Analysis

Table 6 presents the relationship between sexual function and independent variable. As can be seen, the difference was not statistically significant in some of the regression models that controlled for the FSFI score as well as education level, occupation, quality of life (QoL) and anxiety. However, it was found that there is a significant relashionship between sexual dysfunction with depression, infertility and different groups.

**Table 6:** Logistic regression coefficients for sexual function in relation to independent variables

Variable	Beta	SE	*P *value	OR*	(95%CI)
Group					
Group A (HA + PCOM + OD)	1.013	0.673	0.132	2.755	(0.736,10.308)
Group B (HA + OD)	2.139	0.946	0.024	8.490	(1.329,,54.23 1)
Group C (HA + PCOM)	1.287	0.683	0.06	3.623	(0.941,13.829)
Group D (OD + PCOM)	1.213	0.688	0.078	3.362	(0.873,12.946)
Group5 (Control)				1	
HADS					
Anxiety	− 0.011	0.052	0.830	0.989	(0.893,1.095)
Depression	0.189	0.059	0.001	1.208	(1.076,1.356)
Infertility					
Infertility1 (yes)	0.849	0.308	0.006	2.338	(1.277,4.279)
Infertility2 (no)				1	
Education					
Education1 (primary)	0.530	0.416	0.203	1.669	(0.751,3.840)
Education2 (secondary)	0.400	0.336	0.234	1.491	(0.773,2.879)
Education3 (higher)				1	
Occupation					
Occupation1 (housewife)	0.556	0.423	0.181	1.762	(0.768,4.038)
Occupation2 (employed)				1	
Quality of life					
In mental field (MCS)	− 0.017	0.021	0.405	0.983	(0.943,1.024)
In physical field (PCS)	− 0.035	0.024	0.149	0.966	(0.922,1.012)

* Regression logistic analysis

So that depressed subjects are about 1.2 time more likely to have sexual dysfunction than nondepressed subjects. As a result, a significant relashionship is obserwed between infertility and sexual function and infertile women are about 2.3 time likely to have sexual dysfunction. Also about groups, it is found that the chance of developing sexual dysfunction in phenotype B is 8.5 times higher than other phenootypes and controls.

Table 7 compares the variable scores in the fertile and infertile groups. As shown, there is a significant difference in depression score between the fertile and infertile groups.

**Table 7:** Comparison of the variable scores in the fertile and infertile groups

Variable	Fertile (n = 79)	Infertile (n = 163)	P*
	Mean (SD)	Mean (SD)	
FSFI	25.04 ± 3.60	24.58 ± 4.12	0.57
Anxiety	10.10 ± 3.72	10.66 ± 3.98	0.48
Depression	5.48 ± 3.50	7.01 ± 3.76	0.04

FSFI: Female Sexual Function Index

*Values are given as mean SD using Student’s t−test

## Discussion

The aim of this study was to evaluate sexual function, anxiety, depression and QoL in different phenotypes of PCOS compared with healthy fertile women referred to the Infertility Clinic of Arash Hospital in Tehran, Iran.

Polycystic ovary syndrome is one of the most common fertility problems in women, which can lead to many psychological disorders such as anxiety and depression [31].

Numerous studies confirm psychological disorders such as sexual function, anxiety and depression in women with PCOS.

According to the obtained results, in the FSFI questionnaire, there were significant differences in terms of arousal, lubrication, pain and total FSFI between the different phenotypes of PCOS and the health controls (P < 0.05). It was also found that sexual symptoms’ scores in phenotype B were significantly lower as compared with the other phenotypes and the control group, which corresponded to the study of Bazarganipoor et al. [32], who reported more sexual dysfunction in the menstrual irregularities and HA group than in two other phenotypes. These results can be explained by hormonal difference. Because phenotypes A and B were considered as classic PCOS and can possess more menstrual dysfunction, higher androgen and insulin levels, increased rate of insulin resistance and being at higher risk of metabolic syndrome and obesity comparing to non HA-phenotypes [21]. It seems elevated luteinizing hormone (LH) level can cause increased synthesis of androgens and increased circulating androgen levels leads to a variety of virilizing changes including: clitromegaly, hirsutism, acne, alopecia, etc. [33].

However HA can have a prominent role in PCOS diagnosis, the association between levels androgen and sexual function remain inconsistent. Ercan et al. [7] reported a significant negative correlation between the scores of total FSFI and LH, total testosterone and free testosterone, which is consistent with the study of Veras et al. [4], who reported a negative collection between sexual function and total testosterone levels, luteinizing hormone and DHEAS. However, mansson et al. and stovall et al. showed an inverse relationship [9, 34].

The possible meaning of the negative correlation between sexual function and androgen levels might be linked to hirsutism, acne vulgaris and subsequent poor body image, leading to esthetic problems, which may affect the psychosexuality of PCOS patients [4, 8].

Also, irregular menstrual, which is one the parameters used to define PCOS, can manifest in classic phenotypes more than others [21]. Although it may seem that an irregular cycle can impair psychological function, but no association between menstrual irregulation and sexual function has been confirmed [14, 35].

It is known that PCOS have a significant negative impact on women’s QoL. Several studies have confirmed psychological disorders such as sexual function, significant reduction in quality of life, anxiety and depression in women with PCOS [36, 37].

In our study, women with PCOS had significantly lower scores in several subscales and in the MCS, which is consistent with the previously published literature [32, 38–40].

Sanchez et al. [38] identified that all women with PCOS and anovulatory PCOS presented lower score in PCS compared to the controls. Moreover, lower scores were reported for five out of the eight scales (role physical, bodily pain, general health, vitality and role emotional) and no were observed for MCS between women with or without PCOS or its phenotypic subtypes. However, we observed significant differences for general health, social function, role emotional and mental health. Unlike the above study, in our research no significant differences were observed for PCOS between different phenotypes and the controls for PCS. In a nation-wide survey in Germany, using SF-12 scale, Benson et al. [39], found that women with PCOS were at higher risk of common psychiatric disorders such as anxiety, depression or both, which were related to lower HRQL. Another study by Bazarganipoor et al. [32], using SF-36 scale showed, the psychologic dimension was more affected than the physical domain, and psychologic impairment was higher in patients with HA and menstrual irregularities than in the two other phenotypes. This finding is consistent with the results of our study. Moran et al. [40], who compared different PCOS phenotypes based on the National Institute of Health (NIH) criteria (HA and OD), reported poorer HRQL in women with NIH PCOS compared with non-NIH PCOS women. Also, they found there are similar anxiety and depression levels in women with NIH and non-NIHPCOS.

Asena Gokcay et al. [41] indicated that the BDI-II scores of phenotype A were higher than those of phenotype D, and the BAI-II scores of phenotype A were higher than those of phenotype B, C and D. This indicates lower depression scores in the non-hyper androgenic phenotype. Although the causes and precise mechanism underlying the increased risk of depression and anxiety in women with PCOS remain unclear, this can be hypothesized due to the effect of hyperandrogenism that lead to hirsutism and acne, and potential factors such as obesity, insulin resistance may act together [41].

Studies suggest interventions that focus on changes in lifestyles or medical treatments might help to improve QoL in PCOS women [42, 43].

According to our results, these suggested interventions can be appropriate when it comes to phenotypic subtypes- mainly classic PCOS women (phenotype A and B). But the current evidence is, to our knowledge, limited and further interventional research regarding improvement of QoL in different of phenotypes of PCOS is warranted.

Also, the results of the current study showed a significant negative correlation between depression score and total FSFI score, indicating the negative effect of depression on sexual function. Similar results were published by Satyko Kogure et al.; there were negative correlation between HADS-A and HADS-D scores and FSFI total score [44]. This finding is in agreement with the findings of Lakatos et al. [45] and Shahraki et al. [46].

Women may experience emotional conditions such as depression, anxiety and lowered self-esteem that are known causative factors of sexual dysfunction.

Marital distress may be created following the diagnosis of infertility. In these women, unsuccessful treatment attempts are known to be the main risk factor of psychological distress [47].

Infertility is common in PCOS women due to anovulation and oligoovulation [48]. Furthermore, infertility in these women poses a risk for psychiatric disorders.

The relationship between sexual function, anxiety and depression with infertility remains controversial. In a study conducted by Diamond et al. [49], it was stated that sexual function in PCOS women did not differ from those without PCOS, and, similarly, there was no significant difference between sexual function in the women with infertility. Kukur Suna K et al. [50] reported that sexual dysfunction was identical was between the infertile and healthy women, but depression scores were higher in the infertile women than the control group, which is consistent with the results of our study. Moreover, Monga et al. [51] reported no significant difference in sexual function between infertile group and fertile group, but quality of life in the infertile group was lower than that the fertile group. These finding are contrary to the finding of Deniz et al. [52]. They reported that PCOS women with infertility had more problems in all sexual function subscales except arousal, indicating that infertility poses a significant risk of sexual dysfunction in PCOS women.

In contrast to our study, in the study of Davari Tanha et al. [53] total FSFI score scores of all domains were statistically higher in the controls. It is worth noting that infertility affects different aspects of the couples’ lives, including psychological well-being [54].

Depression, anxiety, sexual dysfunction and impaired QoL have been reported in more infertile women comparing with fertile women [51, 53].

By means of the FSFI and core fertility QoL questionnaires, Lo et al. [55] showed that infertile women with sexual dysfunction had lower QoL score. Also Sezgin et al. [56] found that infertile had worse QoL than fertile women.

Depression is the most common psychological problem in women affected by infertility [53].This could be due to unsuccessful treatment of infertility and pressure on women as the sense of powerlessness (45, 57).

There is a polar relationship between depression and infertility treatment because depressed people do not follow treatment and couples seeking treatment become depressed after failure [58].

Furthermore, infertility is a medical situation that has various effects on women’s lives.

The effect of BMI and age on sexual function in women with PCOS is controversial. In this study, BMI did not have any significant effect on sexual function. Consistent with our results, Eftekhar et al.’ study reported that BMI did not have any significant effect on the total sexual function score [59]. Similar results were published by Ferraresi et al. who found no association between BMI and FSFI and reported that both obese and non-obese PCOS women had borderline sexual function scores [8]. Although, sexual function has been reported to be negatively correlated to BMI [5, 34], Naumova et al. [60], noted a marked association of age and BMI with reduced FSFI total scores. However, in the study of Stovall and colleagues demonstrated that increasing BMI was associated with a significant reduction in the orgasm subdomains [9]. Satyko Kogure et al. [44] found overweight and obesity were risk factors for the degree of dissatisfaction.

Also, in this study the present findings revealed a significant relashionship was obserwed between age and sexual function, and with increasing one year of age, the chance of developing sexual dysfunction increased by 8.5%. Similar results were published by Bancroft et al. They found this is distress about the relationship and one’s own sexuality in women aged between 20 and 65 years [61]. There is general agreement in the literature that with increasing age, there is a decline in desire and sexual interest. However, there are reports of these problems declining with age. Laumann’ study showed that anxiety about sexual function decreased with increasing age [62], and, similar results were published by Richters: While anxiety during sex remained constant with age, worrying about attractiveness decreased [63]. Koster et al. [64] found no change in desire with increasing age.

The controversial results documented can explain by the fact that the symptom perception of PCOS varies widely according sociocultural factors [65].

Of the strengths of the present study, this study is one of the first attempts to thoroughly perceive differences between PCOS phenotypes in terms of anxiety, depression, sexual function and QoL with the control group. Moreover, it provided an important opportunity to advance the understanding of the requirement of specific mental health screening for each phenotype. Measurement of androgen levels, ultrasonography and complete examinations were performed for all participants in this study, as well as, both groups of women (PCOS and the controls) were studied simultaneously. However, this study had some limitations. First, it was conducted in a tertiary center. Secondly, we did not collect comprehensive information of the control group. Thirdly, the number of subjects in each phenotype was limited and.

Fourthly, the lack of evidence to explain why only phenotype B had lower score in HADS and QoL, although the phenotypes A and B were considered as classic PCOS and common characteristics. Finally, we had no information about the women’s partners.

We suggest multi-center investigations with larger sample sizes and comprehensive evaluation of male partners and control group in future studies.

## Conclusion

The results of the present study showed significant differences in terms sexual function, depression, anxiety and QoL in different phenotypes of PCOS and compared with the healthy group. The higher prevalence of depression and anxiety, and higher impairment in SF an d QoL suggest that hyperandrogenism may have a causative effect on pathogenesis depression, anxiety and sexual dysfunction in women.

## Data Availability

The datasets used and analyzed during the current study are available from the corresponding author on reasonable request.

## Abbreviations

FSFI: Female sexual function index
HADS: Hospital anxiety and depression scale
M-FG: Modified ferriman gallway
PCOS: Polycystic ovary syndrome
SF-12: Short form health survey
NIH: National institute health
OD: Ovulatory dysfunction
WC: Waist circumference
BMI: Body mass index
HC: Hip circumference
QoL: Quality of life
MW: Mann–Whitney
FSD: Female sexual dysfunction
HA: Hyper androgenic
